# Supplementary figures and images for: PURA syndrome-causing mutations impair PUR-domain integrity and affect P-body association
Source: eLife. 2024 Apr 24;13:RP93561. doi: 10.7554/eLife.93561 (PMC11042805; doi:10.7554/eLife.93561)

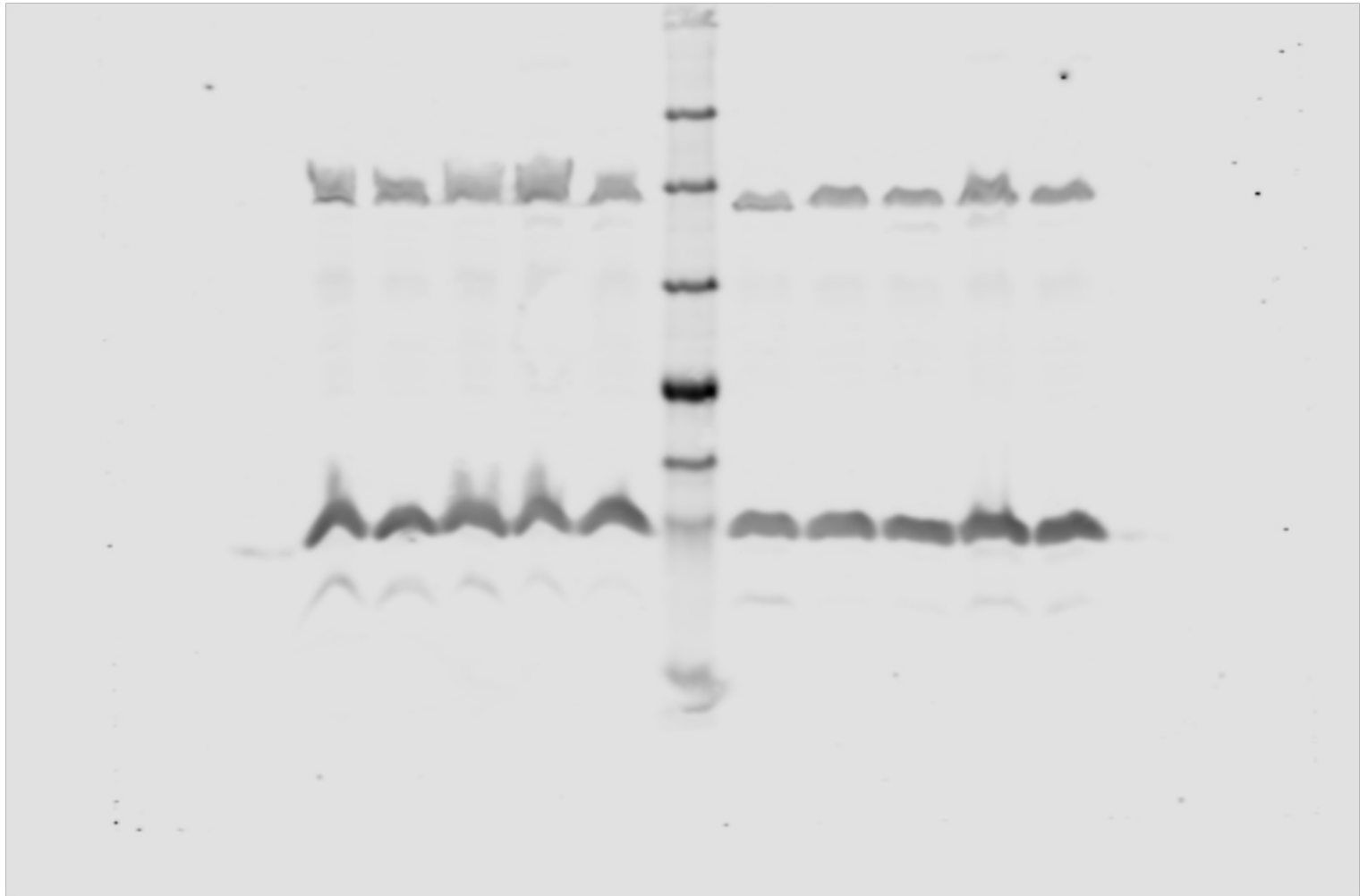

Supplement: Figure 1—figure supplement 1—source data 1. [file elife-93561-fig1-figsupp1-data1.pdf]

Flag-hsPURA F233del  
Flag-hsPURA K97E  
Flag-hsPURA WT  
Flag-hsPURA I206F  
Flag-hsPURA m17

[kDa]

58

46

32

25

22

17

← hsPURA

← H3

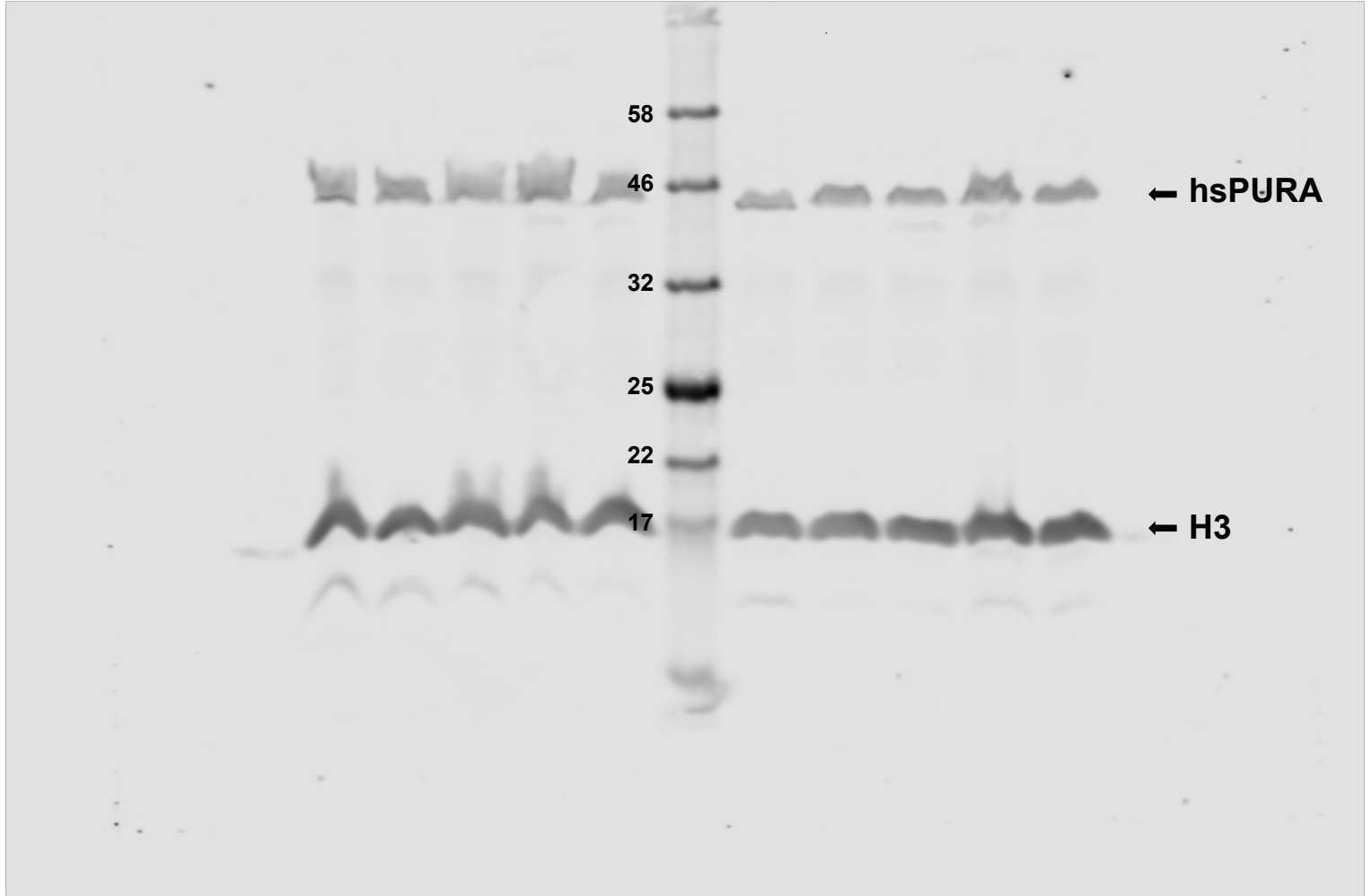

Supplement: Figure 1—figure supplement 1—source data 2. [file elife-93561-fig1-figsupp1-data2.zip › Figure_1-Figure_Supplement_1A-source_data_2.pdf]

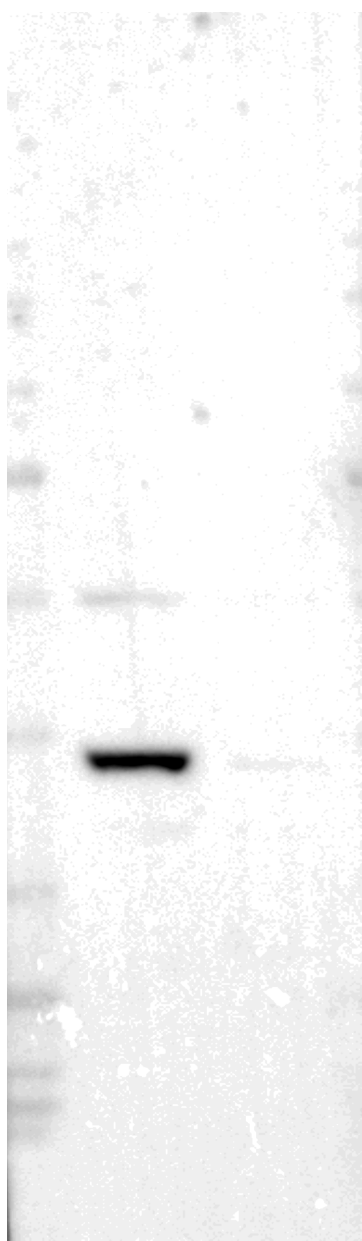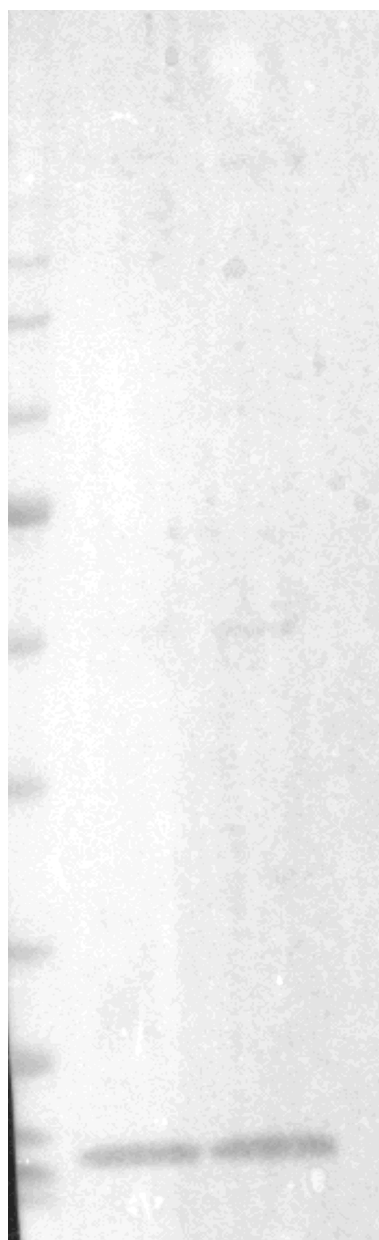

Supplement: Figure 1—figure supplement 3—source data 1. — Western blot of control (scrambled sirRNA) and PURA knockdown HeLa cells anti-PURA and H3 as housekeeping gene – uncropped, raw images. [file elife-93561-fig1-figsupp3-data1.zip › Figure_1-Figure_Supplement_3A-source_data_1.pdf]

*anti-PURA*

**CTRL KD**

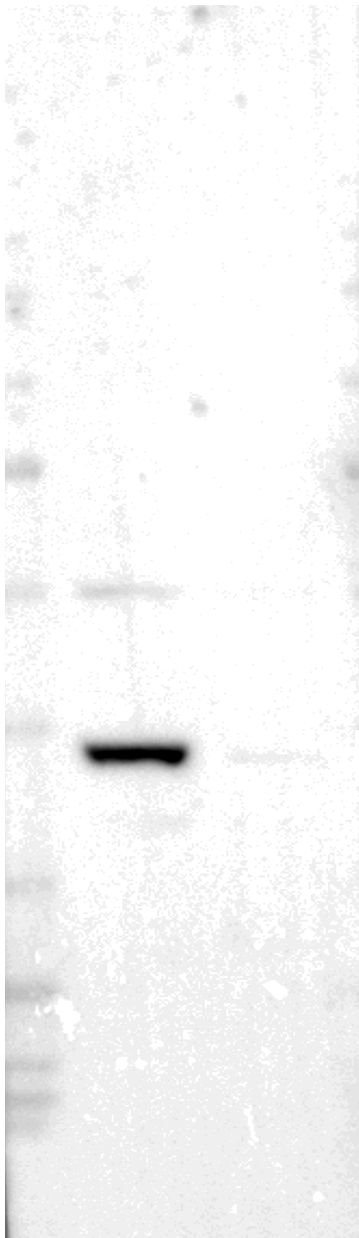

▷ **PURA**  
~35 kDa

*Repeat 1*

*anti-H3*

**CTRL KD**

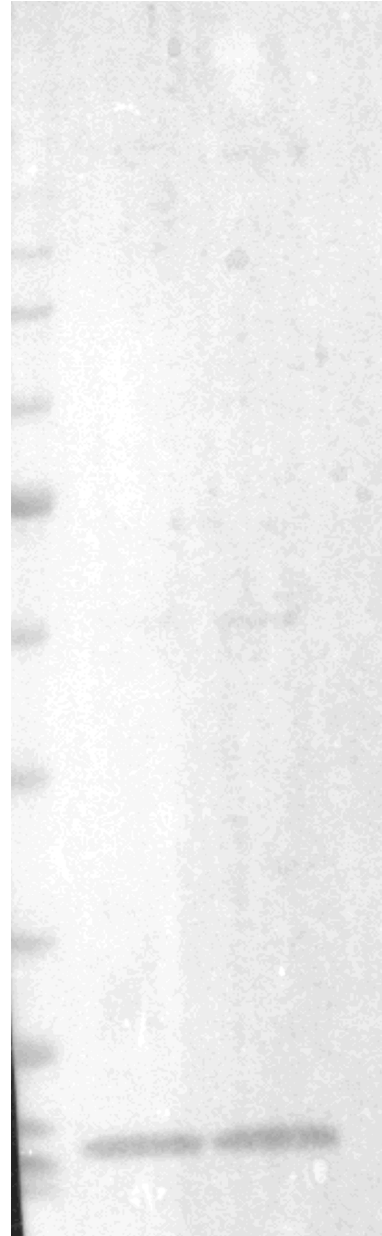

▷ **H3**  
~18 kDa

*Repeat 1*

Supplement: Figure 1—figure supplement 3—source data 1. — Western blot of control (scrambled sirRNA) and PURA knockdown HeLa cells anti-PURA and H3 as housekeeping gene – uncropped, raw images. [file elife-93561-fig1-figsupp3-data1.zip › Figure_1-Figure_Supplement_3A-source_data_2.pdf]

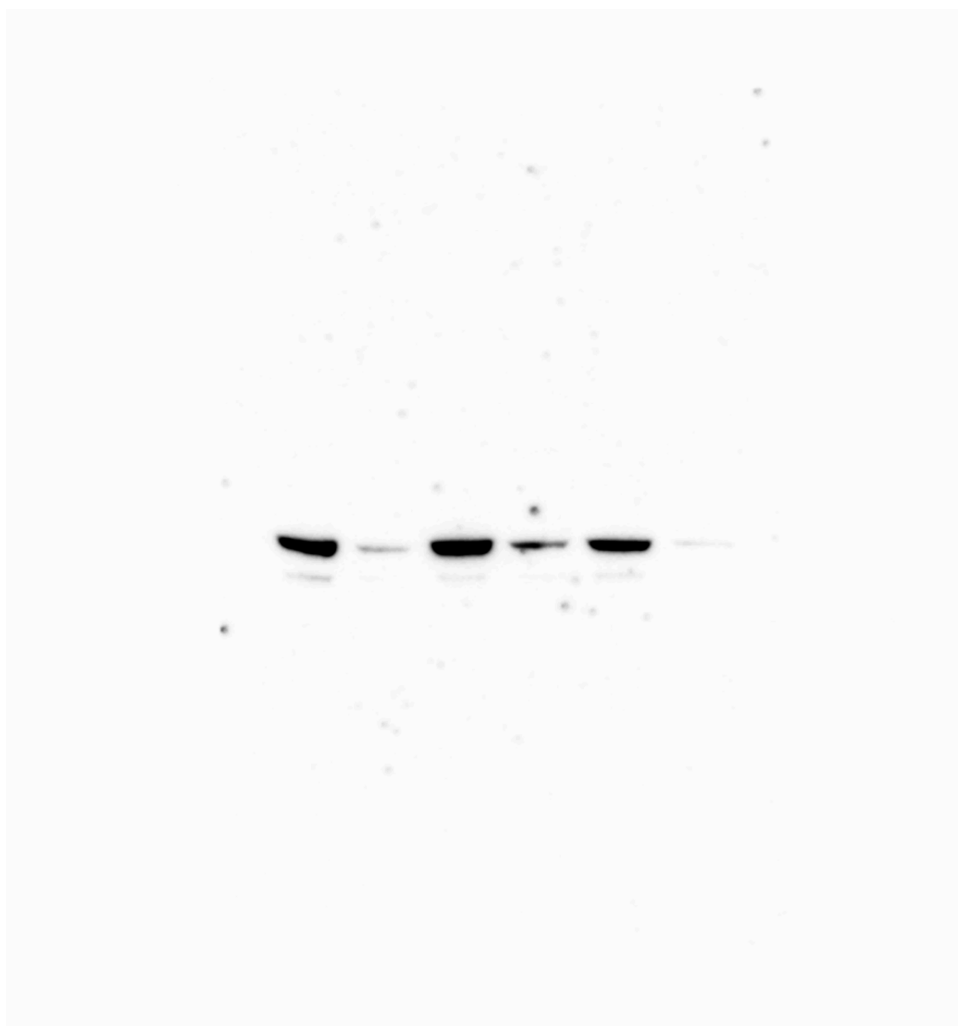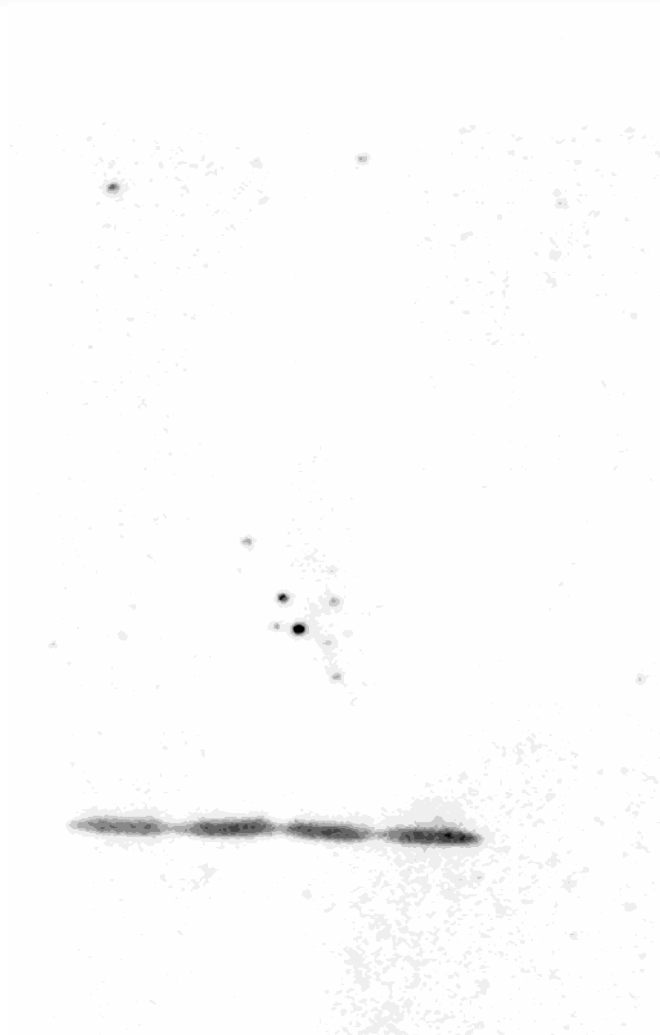

Supplement: Figure 1—figure supplement 3—source data 2. — Western blot of control (scrambled sirRNA) and PURA knockdown HeLa cells anti-PURA and H3 as housekeeping gene – uncropped, labeled images. [file elife-93561-fig1-figsupp3-data2.zip › Figure_1-Figure_Supplement_3C-source_data_1.pdf]

*anti-PURA*

CTRL KD CTRL KD

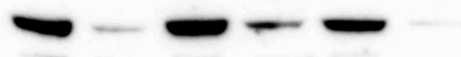

▷ **PURA**  
~35 kDa

*Repeat 2&3*

*anti-H3*

CTRL KD CTRL KD

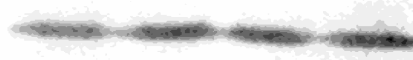

▷ **H3**  
~18 kDa

*Repeat 2&3*

Supplement: Figure 1—figure supplement 3—source data 2. — Western blot of control (scrambled sirRNA) and PURA knockdown HeLa cells anti-PURA and H3 as housekeeping gene – uncropped, labeled images. [file elife-93561-fig1-figsupp3-data2.zip › Figure_1-Figure_Supplement_3C-source_data_2.pdf]
