# Supplementary material for: PURA syndrome-causing mutations impair PUR-domain integrity and affect P-body association
Source: eLife. 2024 Apr 24;13:RP93561. doi: 10.7554/eLife.93561 (PMC11042805; doi:10.7554/eLife.93561)

EMSA *hs*PURA III

Scan date & time: 2021.07.30 11:35:55
Export date & time: 2021.07.30 12:13:08
Instrument S/N: 86350374
Software version: 2.0.0.6
Pixel size: 100 micrometer
Scan speed: slow

File name: III_run3
[Cy5], PMT: Multi-alkali 953V

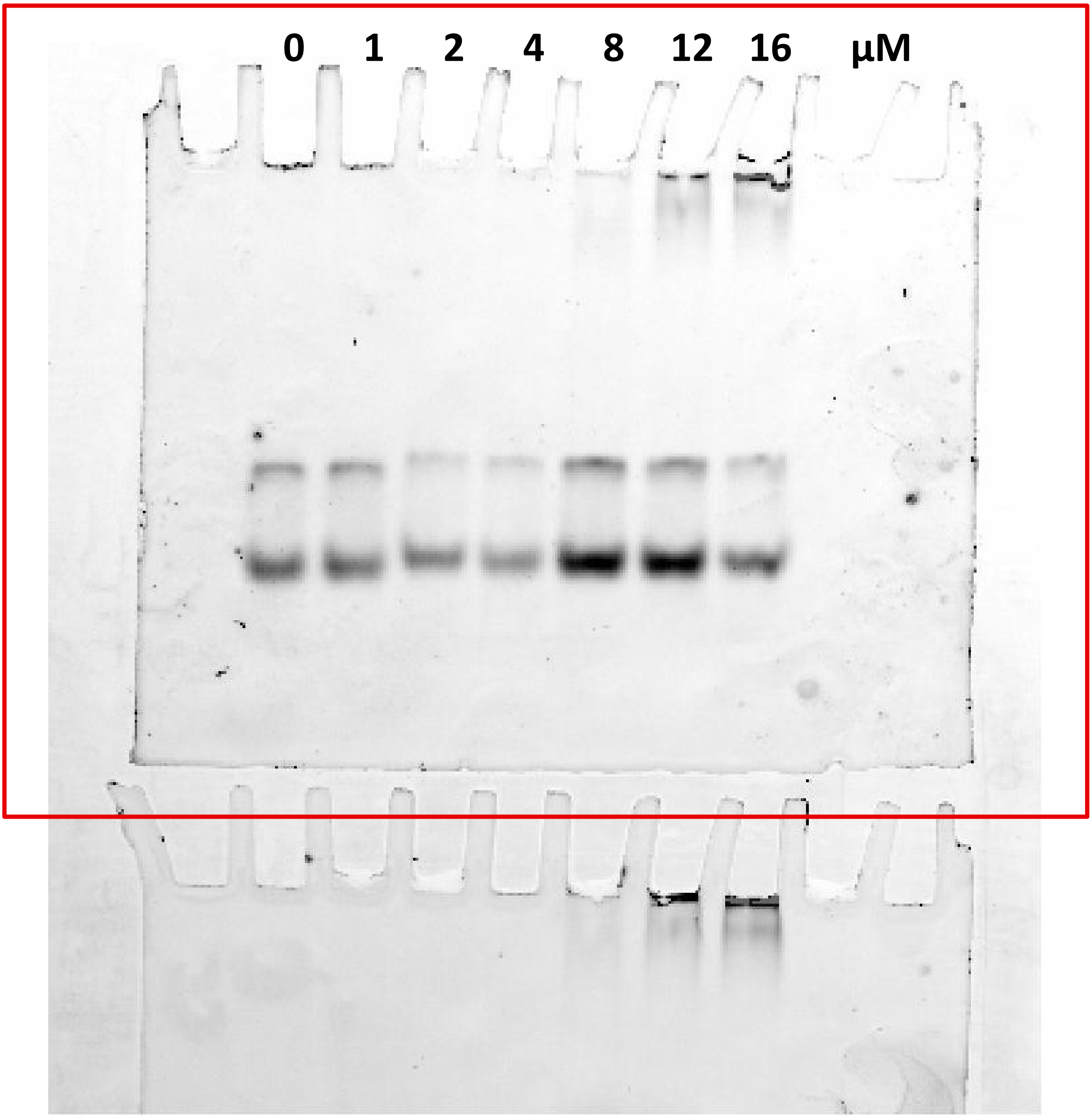

Supplement: Figure 3—source data 2. — Uncropped, labeled EMSA gel image for hsPURA III. [file elife-93561-fig3-data2.zip › Figure_3B-source_data_2.pdf]
