# Supplementary material for: PURA syndrome-causing mutations impair PUR-domain integrity and affect P-body association
Source: eLife. 2024 Apr 24;13:RP93561. doi: 10.7554/eLife.93561 (PMC11042805; doi:10.7554/eLife.93561)

# EMSA *hs*PURA FL

Scan date & time: 2021.08.03 12:11:21
Export date & time: 2021.08.03 13:11:24
Instrument S/N: 86350374
Software version: 2.0.0.6
Pixel size: 100 micrometer
Scan speed: slow

File name: K97_WTFL
[Cy5], PMT: Multi-alkali 696V

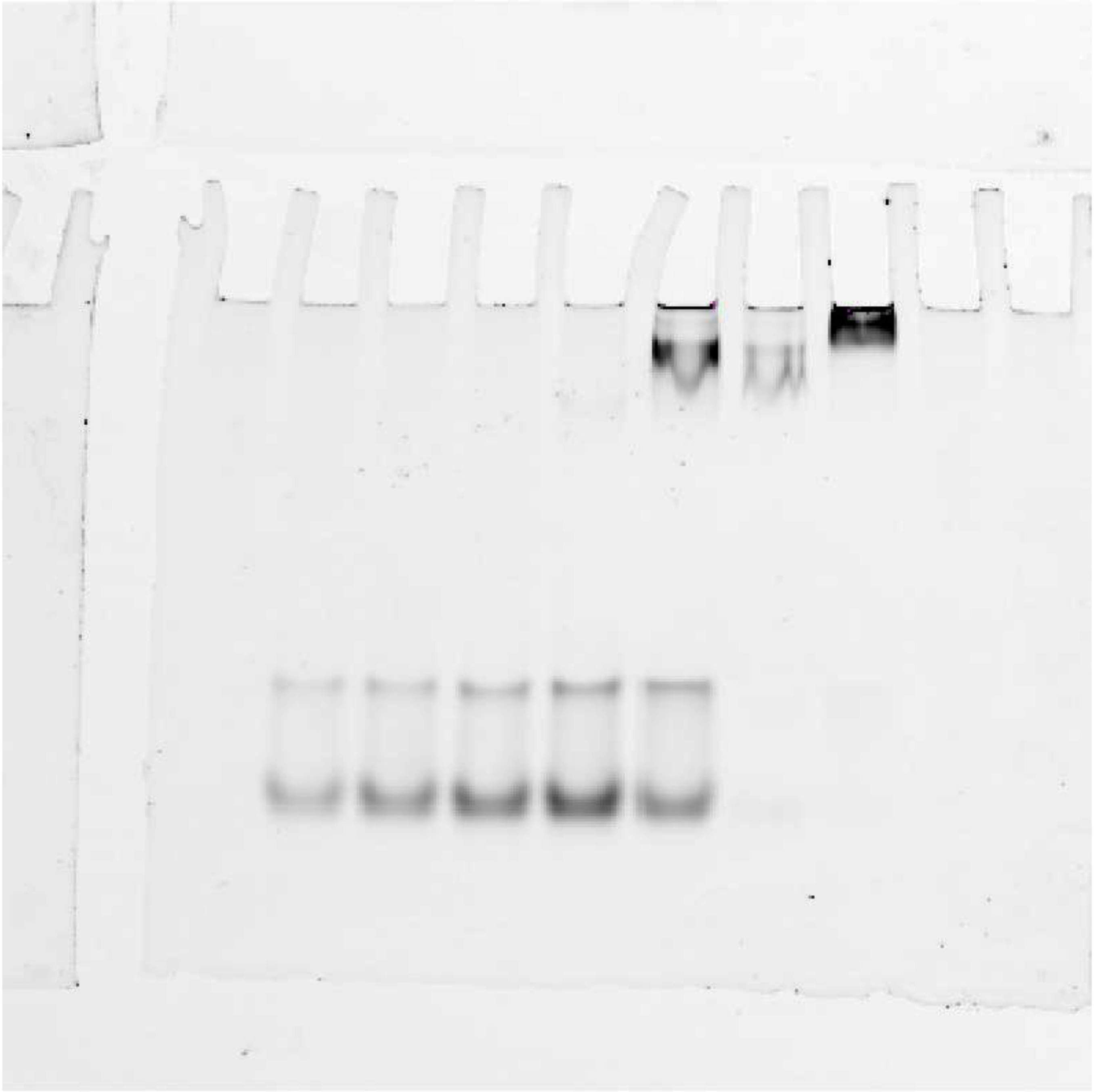

Supplement: Figure 3—source data 3. — Uncropped, raw EMSA gel image for hsPURA FL. [file elife-93561-fig3-data3.zip › Figure_3B-source_data_3.pdf]
