# Supplementary material for: PURA syndrome-causing mutations impair PUR-domain integrity and affect P-body association
Source: eLife. 2024 Apr 24;13:RP93561. doi: 10.7554/eLife.93561 (PMC11042805; doi:10.7554/eLife.93561)

EMSA *hs*PURA I-II R140P

Scan date & time: 2021.07.05 15:35:23
Export date & time: 2021.07.05 16:29:42
Instrument S/N: 86350374
Software version: 2.0.0.6
Pixel size: 100 micrometer
Scan speed: slow

File name: R140P_run1
[Cy5], PMT: Multi-alkali 794V

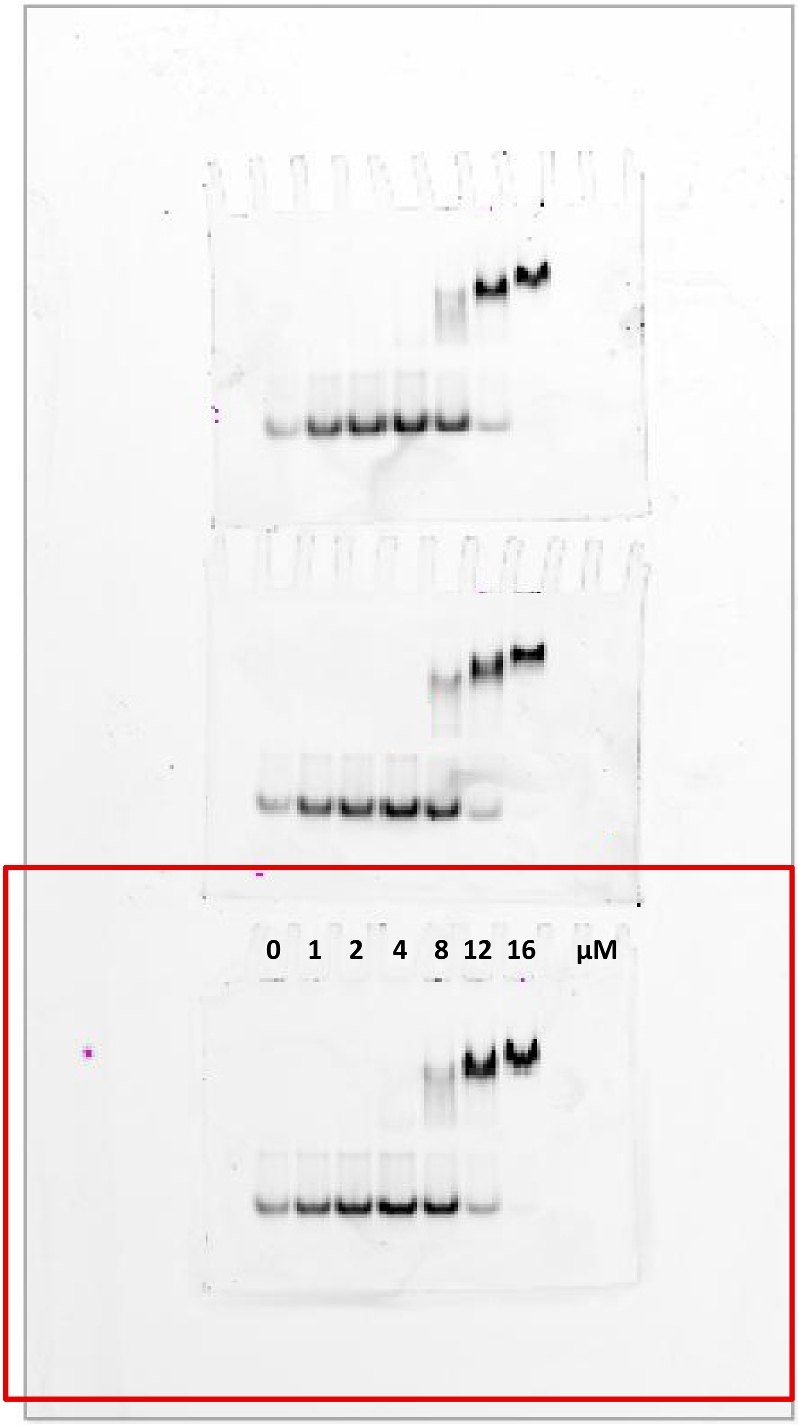

Supplement: Figure 4—source data 4. — Uncropped, labeled EMSA gel image for hsPURA I–II R140P. [file elife-93561-fig4-data4.zip › Figure_4C-source_data_4.pdf]
