# Supplementary material for: PURA syndrome-causing mutations impair PUR-domain integrity and affect P-body association
Source: eLife. 2024 Apr 24;13:RP93561. doi: 10.7554/eLife.93561 (PMC11042805; doi:10.7554/eLife.93561)
Supplement: Supplementary file 1. — (A) Summary of the experiments in this study for the selected human PURA variants. (B) Data collection and refinement statistics for crystal structures of human PURA variants. Values in parentheses are for the highest resolution shell. [file elife-93561-supp1.docx]

Supplementary File 1a.

| **Protein construct** | **Purification** | **Structure** | **EMSA** | **dsDNA Strand separation** | **NanoBRET** | **Stress Granules** | **P-bodies** |
| --- | --- | --- | --- | --- | --- | --- | --- |
| PURA I-II WT | 🗸 | 🗸 | 🗸 | 🗸 | 🗸 | 🗴 | 🗴 |
| PURA I-II K97E | 🗸 | 🗸 | 🗸 | 🗸 | 🗴 | 🗴 | 🗴 |
| PURA I-II R140P | 🗸 | 🗸 | 🗸 | 🗸 | 🗴 | 🗴 | 🗴 |
| PURA I-II R199P | 🗴 | 🗴 | 🗴 | 🗴 | 🗴 | 🗴 | 🗴 |
| PURA I-II I206F | 🗴 | 🗴 | 🗴 | 🗴 | 🗴 | 🗴 | 🗴 |
| PURA I-II m11 | 🗸 | 🗴 | 🗸 | 🗸 | 🗴 | 🗴 | 🗴 |
| PURA III WT | 🗸 | 🗸 | 🗸 | 🗸 | 🗴 | 🗴 | 🗴 |
| PURA III F233del | 🗴 | 🗴 | 🗴 | 🗴 | 🗴 | 🗴 | 🗴 |
| PURA III R245P | 🗴 | 🗴 | 🗴 | 🗴 | 🗴 | 🗴 | 🗴 |
| PURA I-III WT | 🗴 | 🗴 | 🗴 | 🗴 | 🗸 | 🗴 | 🗴 |
| PURA I-III F233del | 🗴 | 🗴 | 🗴 | 🗴 | 🗸 | 🗴 | 🗴 |
| PURA I-III R245P | 🗴 | 🗴 | 🗴 | 🗴 | 🗸 | 🗴 | 🗴 |
| PURA FL WT | 🗸 | 🗴 | 🗸 | 🗸 | 🗴 | 🗸 | 🗸 |
| PURA FL K97E | 🗴 | 🗴 | 🗴 | 🗴 | 🗴 | 🗸 | 🗸 |
| PURA FL I206F | 🗴 | 🗴 | 🗴 | 🗴 | 🗴 | 🗸 | 🗸 |
| PURA FL F233del | 🗴 | 🗴 | 🗴 | 🗴 | 🗴 | 🗸 | 🗸 |
| PURA FL m11 | 🗴 | 🗴 | 🗴 | 🗴 | 🗴 | 🗸 | 🗸 |

Supplementary File 1b.

| **Data collection** | | | | |
| --- | --- | --- | --- | --- |
| Protein | PURA I-II | PURA I-II K97E | PURA I-II R140P | PURA III |
| PDB ID | 8CHT | 8CHU | 8CHV | 8CHW |
| Beamline | PETRA III DESY P11 | SLS PXIII X06DA | SLS PXIII X06DA | SLS PXIII X06DA |
| Wavelength | 1.033100 | 1.000029 | 1.000040 | 1.000040 |
| Space group | *P*2_1_ | *I*2_1_2_1_2_1_ | *P*2_1_ | *P*2_1_2_1_2 |
| Cell dimensions  *a*, *b*, *c* (Å), *β* (°) | 64.92, 58.14, 81.94,  *β* = 100.65 | 40.02, 77.32, 225.38 | 63.31, 57.58, 84.89, *β* = 102.33 | 56.76, 75.64, 31.39 |
| No. of molecules per AU | 4 | 2 | 4 | 2 |
| Resolution (Å) | 50-1.95  (2.00-1.95) | 50-2.45  (2.52-2.45) | 50-2.15  (2.28-2.15) | 50-1.70  (1.74-1.70) |
| *I* / σ*I* | 12.1 (2.2) | 12.1 (1.28) | 7.6 (0.9) | 19.5 (2.4) |
| CC (1/2) | 99.8 (74.3) | 99.8 (61.9) | 99.7 (58.3) | 100 (88.7) |
| Completeness (%) | 98.0 (96.9) | 99.9 (99.5) | 99.4 (97.7) | 100 (99.9) |
| Redundancy | 6.9 (7.0) | 6.5 (6.6) | 3.5 (3.5) | 6.4 (6.7) |
| **Refinement** | | | | |
| Resolution (Å) | 50-1.95 | 39.43-2.45 | 50-2.15 | 37.85-1.7 |
| No. reflections | 43,138 | 13,362 | 32,410 | 15,494 |
| *R*_work_ / *R*_free_ (%) | 17.4/23.1 | 20.2/27.2 | 20.9/28.2 | 17.6/22.4 |
| No. atoms |  |  |  |  |
| Protein | 4,659 | 2,287 | 4,597 | 1,108 |
| Water | 314 | 87 | 197 | 132 |
| Other | 16 | 5 | 0 | 34 |
| *B*-factor overall | 26.2 | 61.0 | 52 | 26.4 |
| R.m.s. deviations |  |  |  |  |
| Bond lengths (Å) | 0.025 | 0.009 | 0.006 | 0.010 |
| Bond angles (°) | 2.31 | 1.65 | 1.46 | 1.57 |
| Ramachandran plot |  |  |  |  |
| Most favored (%) | 97 | 96 | 95 | 96 |
| Additional allowed (%) | 3 | 4 | 5 | 4 |
